# Is intracouple assisted reproductive technology an option for men with large-headed spermatozoa? A literature review and a decision guide proposal

**DOI:** 10.1186/s12610-016-0035-6

**Published:** 2016-07-08

**Authors:** Bruno Guthauser, Xavier Pollet-Villard, Florence Boitrelle, Francois Vialard

**Affiliations:** Laboratoire de Biologie de la Reproduction, Hôpital Tenon, Paris, France; Centre d’AMP Nataliance, Laboratoire Medibio, Saran, France; GIG, EA7404, Université Versailles St Quentin en Yvelines, Montigny le Bretonneux, France; Unité de Biologie de la Reproduction, CHI de Poissy St Germain, Poissy, France

**Keywords:** Macrocephalic sperm head syndrome, Large-headed spermatozoa, Assisted reproductive technology outcomes, Fluorescence *in situ* hybridization, Sperm head measurement, Syndrome des spermatozoïdes macrocéphales, Spermatozoïdes macrocéphales, Issue des aides médicales à la procréation

## Abstract

Although the presence of spermatozoa with an abnormally large head is rare, it is associated with low fertility or even total infertility. We reviewed the literature on assisted reproductive technology (ART) strategies and outcomes for men with large-headed spermatozoa. We also discuss additional analyses that can usefully characterize sperm defects and help with the choice between intra-couple ART and insemination with donor sperm. Lastly, we propose a classification for cases of large-headed spermatozoa.

## Résumé

La présence de spermatozoïdes macrocéphales est généralement associée à une hypofertilité ou une infertilité. Nous présentons une revue de la littérature concernant les stratégies de prise en charge en aide médicale à la procréation et leurs issues lorsque l’analyse du sperme met en évidence la présence de spermatozoïdes macrocéphales. Nous discutons également les examens complémentaires permettant de mieux caractériser ces anomalies spermatiques et de choisir entre l’aide médicale à la procréation avec ou sans donneur de sperme. Enfin nous proposons ici une classification pour les cas de spermatozoides macrocephales avec pour les 6 types définis une proposition de prise en charge.

## Background

Large-headed spermatozoa are defined as those with a length > 4.7 μm and a width > 3.2 μm [1]. A number of different terms have been used to refer to these spermatozoa, including “macrocephalic sperm” [2–5], “megalohead” spermatozoa [6] “enlarged-head spermatozoa” or “enlarged forms” [7–9], “macronuclear spermatozoa” [10] and “large head spermatozoa” [11]. Men with large-headed spermatozoa (who may have a normal sperm count *per se*) account for less than 1 % of cases of severe male infertility. The condition is associated with poor fertility or even complete infertility. Accordingly, in the past 20 years, very few babies have been fathered by men with large-headed spermatozoa as part of assisted reproductive technology (ART) programmes - raising the question of whether donor sperm should be used in this context. Here, we review the scientific literature on ART programmes and outcomes for men with large-headed spermatozoa. We also discuss the potential value of additional analyses in the decision-making process for men with large-headed spermatozoa. Lastly, given that the case reports range from the classic scenario (macrocephalic sperm head syndrome, MSHS, in which nearly all spermatozoa have large heads and multiple flagella) to those featuring less severe sperm abnormalities, we propose a new classification that we hope will enable physicians to help their patients to choose between intracouple ART and insemination with donor sperm.

## Methods

We performed a systematic review of the relevant literature, in accordance with the PRISMA guidelines. In PubMed database we used the following terms: “macrocephalic sperm”, “enlarged head spermatozoa”, macronuclear spermatozoa”, “sperm head measurements”, large-head spermatozoa”. Only studies with a fully described sperm morphology including photographies or precise sperm characteristics were considered.

### Literature review (Table **1**)

**Table 1:** Reports of patients with large-headed spermatozoa and the associated ART decisions and outcomes

Reference	Number of patients	Spermatozoa with		Description of semen			Spermatozoa with aneuploidies	Additive analysis	Status of intracouple ART	Outcome (s) for ART programmes with the patient’s sperm
		Large heads	Multiple flagella	Sperm count (10^6^/ml)	Motility (%)	Atypical form (%)				
Yurov et al., 1996 [11]	1	40 %	/	1.0	1 %	97 %	100 %	/	ICSI: 5 cycles	no pregnancies
In’t veld et al., 1997 [8]	1	100 %	1 to 3 flagella	15.0			100 %	Head area 25.4+/−4 μm^2^	ICSI: 1 cycles	15 oocytes, 2 embryos transferred, no pregnancies
Weissenberg et al., 1998 [14]	1	100 %		10.3	23.7 %		99.2 %	/	ICSI: 2 cycles	14 oocytes, 2 embryos transferred, no pregnancies
Kahraman et al., 1999 [6]	17	68.5 %		3.8	4.7 %	0 %	not determined	/	ICSI: 22 cycles	Fertilization rate: 43 %
										9 embryo transfers
										2 pregnancies and 3 live births
Viville et al., 2002 [40]	1	64 % with multiple flagella		2.0	<5 %		89.2 %	/	ICSI: 3 cycles	No pregnancies.
Benzaken et al., 2001 [3]	1	100 %	72 %	2.0	<5 %		100 %	/	Not performed	/
Devillard et al., 2002 [17]	3	100 % (irregular shape)	30 %	3.2	25 %		100 %	/	Not performed	/
		>95 %	/	30.0	reduced		100 %			
		100 %	/	10.0	reduced		100 %			
Lewis-Hones et al., 2003 [15]	3	46 %	72 %	Oligo-asthenospermia		100 %	100 %	/	Not performed	/
		60 %	49 %				46 %			
		54 %	50 %				82.5 %			
Vicari et al., 2003 [18]	3	54.3 %	/	14.4 %	14 %	100 %	69.5 %	/	Not performed	/
		18.9 %		3.7 %	13 %		5.2 %			
		26.5 %		0.25 %	8 %		13.1 %			
Kahraman et al., 2004 [31]	21	predominantly macrocephalic and pinhead spermatozoa		6.7	14.1 %	/	/	/	ICSI and PGD, 23 cycles	Implantation rate: 25 %
										Pregnancy rate: 33 %
										Abortion rate: 14.3 %
	52			11.9	17 %				ICSI (no PGD), 60 cycles	Implantation rate: 12.3 %
										Pregnancy rate: 27.8 %
										Abortion rate: 46.7 %
Guthauser et al., 2006 [4]	2	91 % with multiple flagella		30.4	0 %	100 %	100 %, 95 % for spermatozoa with normal-sized heads		Not performed	/
		82 % with multiple flagella		3.6			100 %, 90 % for spermatozoa with normal-sized heads			
Achard et al., 2007 [10]	4	29.5 %	/	8.2	32 %	70 %	51.4 %	/	ICSI, 7 cycles	Fertilisation rate: 65.6 %
		22 %	/	6.2	33 %	69 %	43.6 %			
		49.7 %	/	1.3	7 %	91.5 %	71.7 %			One live birth (for patient 1)
		19 %	/	1.7	9 %	96 %	25.6 %			
Perrin et al., 2008 [16]	1	62 %	54 %	2.8	0 %	100 %	99.3 %	/	Not performed	/
Guthauser et al., 2011 [7]	1	47 %	0 %	28.3	8 %	98 %	3 %	Uncondensed chromatin:46 %	ICSI	Lost to follow-up
Ben Khelifa et al., 2011 [9]	2	100 %	28 %	0.9	8 %	MAI: 3.5	/	/	ICSI, 6 cycles	Fertilization rate: 17.0 %
		100 %	52 %	0.8	7 %	MAI: 3.6			ICSI, 5 cycles	
										No pregnancies
Guthauser et al., 2013 [26]	1	12 %, Normally shaped	0 %	89	40 %	80 %	78 %, 68 % for spermatozoa with normal-sized heads	Uncondensed chromatin: 11 %	Not performed	/
Shimizu et al., 2012 [5]	1	“Almost all” (exact % not specified)		0.2	25 %	99.7 %	Not determined		ICSI	One ICSI cycle, one live birth

Studies with sample sizes evaluating the relationship between the percentage of spermatozoa with large heads, multiple flagella, semen characteristics, percentage of spermatozoa with aneuploidies, ART (assisted reproductive technology) performed, and outcomes

The first clinical report of spermatozoa with an abnormally large head and multiple flagella (MSHS) was published almost 40 years ago [12]. A further six cases were described over the following years [13]. Since then, a wide range of clinical contexts has been reported on. The percentage of large-headed spermatozoa in the semen varies; it can be very high (and even 100 %) in some patients and low to moderate in others (Table 1). These clinical variations differ from the original case report and are discussed below.

### Cases with a high percentage of large-headed spermatozoa with an uneven shape and multiple flagella: MSHS

A high proportion of irregularly shaped, multi-tailed spermatozoa (see photograph 1 in Table 2) is associated with severe male infertility. High rates of polyploidy and aneuploidy have been described in these cases [2–4, 14–16]. Most studies have highlighted a highly abnormal sperm chromosomal content, which suggests a high genetic risk for the conceptus. Viville et al. reported a patient in whom 64 % of the spermatozoa had a large head and multiple flagella; in a fluorescence *in situ* hybridization (FISH) analysis, 89.2 % of the spermatozoa were found to be aneuploid or polyploid [2]. Benzaken et al. described a case in which all the spermatozoa were large-headed and polyploid; ICSI was therefore contraindicated [3]. Devillard et al. found for 3 patients with large headed sperm and multiflagella a polyploidy chromosomal constitution, and ICSI was not recommended nor performed [17]. In 2006, we reported on two patients in whom respectively 91 % and 82 % of the spermatozoa were large-headed. Interestingly, 95 % (27/28) and 90 % (46/51) of the few spermatozoa with a normal-sized head had an abnormal chromosomal content (according to a FISH analysis of chromosomes X, Y and 18). Insemination with donor sperm was therefore recommended [4]. Lastly, Perrin et al. studied the chromosomal status of large-headed spermatozoa with multiple flagella and decided not to recommend ART with the patient’s sperm [16].

**Table 2:** Suggested additional analyses performed for ART decision

Sperm phenotype		Large head with irregular shape and multiple flagella		Large head with a regular shape	
Light microscopy		(Photo 1)		(Photo 2)	
Sperm head measurement (200 cells, CASMA)		Determination of the percentage of spermatozoa with a large head and the percentage with a normal-sized head		Determination of the percentage of spermatozoa with a large head	
FISH analysis on raw semen (with X/Y/18 probes) Photo 3		100 % aneuploidy, Donor sperm or adoption	<100 % aneuploidy	A normal percentage of spermatozoa with abnormal chromosome content	An elevated percentage of spermatozoa with abnormal chromosome content
FISH analysis of selected spermatozoa with a normal-sized head Photo 4		/	Percentage of aneuploid spermatozoa Low sperm aneuploidy?	/	Percentage of aneuploid spermatozoa Low sperm aneuploidy?
AURKC screening		Mutation detected: stop ART with patient’s sperm		Optional	
Chromatin condensation rate in whole sperm		/	/	Within the normal range	Not within the normal range
Chromatin condensation assay on spermatozoa with a normal-sized head (sperm measurement + aniline blue staining		/	/	/	Chromatin condensation rate for spermatozoa with a normal-sized head
Genetic counselling		Decision on ART according to the results of an aneuploidy assessment, AURKC mutation screening and the couple’s history of fertility/infertility		Decision on ART according to the sperm chromatin condensation rate, the FISH result on whole sperm and (in some cases) the percentage of spermatozoa with a normal-sized head and the couple’s history of fertility/infertility	

Decision on ART according to sperm phenotype, light microscopy examination, FISH analysis, AURC screening, chromatine condensation

### Cases with a moderate percentage of large-headed spermatozoa

Yurov et al. described a patient in whom 40 % of spermatozoa had abnormally large heads. However, all the spermatozoa had a regular shape and normal flagella (see photograph 2 in Table 1) [11]. Similarly, Vicari et al. reported on three patients in whom respectively 54 %, 19 %, and 26 % of the spermatozoa had an abnormally large head [18]. Achard et al. published a report on four cases, in whom respectively 19 %, 22 %, 29 % and 49 % of the spermatozoa had a large head. The sperm head’s shape was irregular in three of the patients and regular in one. In line with the proportions of large-headed spermatozoa, a FISH analysis of three chromosomes revealed that respectively 25.6 %, 43.6 %, 51.4 % and 71.7 % of the spermatozoa were aneuploid or polyploid [10].

### Intra-couple ART and pregnancy outcomes

Over the past 20 years, 18 publications have reported on a total of 124 patients with large-headed spermatozoa. Sperm chromosome analysis was performed in almost all of these studies; the FISH results and intra-cytoplasmic sperm injection (ICSI) outcomes are summarized in Table 1. One hundred and one patients (in 9 different studies) entered intracouple ICSI programmes, whereas ICSI was contraindicated for 23 patients in 8 studies. A total of 111 ICSI cycles were performed but 94 % of these attempts failed - even when embryos were obtained (Table 1). Ben Khelifa et al. [9] reported the performance of 11 ICSI cycles with sperm from 2 patients. All the spermatozoa were large-headed and respectively 28 % and 52 % of the spermatozoa had multiple flagella. 26 embryos were transferred but none resulted in a pregnancy.

The 111 ICSI cycles resulted in 5 live births. Kahraman et al. reported on a series of 22 ICSI cycles. The fertilization rate (43 %) and the pregnancy rate (9 %) per cycle were low. Nineteen embryo transfers resulted in 2 pregnancies and 3 live births [6]. Achard et al. reported on 7 ICSI cycles in 4 patients with a moderate proportion of large-head spermatozoa. The sole reported pregnancy resulted in a live birth [10]. In 2012, Shimizu et al. reported the birth of a healthy baby after ICSI for a patient with MSHS. They wrote that “*almost all spermatozoa had enlarged heads and multiple flagella*”, although the percentage was not specified and additional assessments (such as a FISH analysis) were not undertaken. ICSI was nevertheless performed using spermatozoa with an “*almost normal form*” [5].

### The potential value of additional analyses of sperm with large-headed spermatozoa (Table 2)

Additional assessments may prove useful for better characterizing sperm defects, assisting with the decision-making process (i.e. initiation of ART with the patient’s sperm or with donor sperm) and choosing the best spermatozoon for injection if ICSI with the patient’s sperm is undertaken.

### Sperm head morphometry with light microscopy

In cases where all the spermatozoa have a large head, an irregular shape and multiple flagella (as initially described by Nistal et al. in 1977 [12]), MSHS is easy to diagnose. In contrast, distinguishing between normal-headed spermatozoa and normally shaped spermatozoa with enlarged head can be more difficult. In such a case, the precise measurement of the head’s length and width (at least) with a micrometre eyepiece is recommended [1]. The sperm head’s dimensions (i.e. length, width, surface area and perimeter) are useful for detecting slight variations in size. The use of computer-assisted sperm morphometry analysis (CASMA) can reduce intra- and inter-operator variability. In animal models, CASMA is commonly used to provide objective sperm head measurements [19–23]. Hingst et al.’s study of cat semen found that an abnormally large head size was associated with incompletely condensed chromatin [19]. Casey et al. reported that subfertile stallions had a higher sperm head surface area than fertile stallions [24]. Lastly, Dahlbom et al. found two populations of dog spermatozoa with different head sizes [20].

Similarly, CASMA has been used to evaluate the fertility of men with large-headed spermatozoa [7, 25, 26]. However, it is important to note that head dimension parameters depend on the staining methods used. When compared with the classic modified Papanicolaou procedure, rapid staining procedures (such as Hemacolor® or DiffQuick® methods) cause the spermatozoa to swell and thus alter the head’s morphology. A recent review reported significant inter-method differences, with sperm head surface areas ranging from 9.45 ± 1.35 μm^2^ (for Papanicolaou staining) to 18.83 ± 1.37 μm^2^ (for Hemacolor® staining) [27]. Given the heterogeneity and absence of standardization of procedures available for sperm head measurement after staining, one must be very cautious when establishing size thresholds. Innovative CASMA technologies based on fluorescence or phase contrast microscopy may facilitate standardization [27].

### Sperm chromatin condensation assays

In a study of bull spermatozoa, Ferrari et al. showed that an abnormally high nuclear volume was associated with altered chromatin condensation [21]. Revay et al. also reported an alteration of chromatin condensation in large-headed bull spermatozoa [28]. Sperm chromatin condensation (using the aniline blue assay) might be useful in cases featuring large-headed but regularly shaped spermatozoa [7]. It has recently been shown that large-headed spermatozoa present a high degree of non-condensed chromatin [29]. Although the utility of sperm chromatin condensation assays is still subject to debate, these tests might provide a more accurate prognosis for patients with a high percentage of large-headed spermatozoa (Table 3).

**Table 3:** Proposed classification for semen containing large-headed spermatozoa and ART possibilities decision

Light microscopy for large-headed spermatozoa	Proposal of percentage of spermatozoa with large heads	Proposal of FISH on selected spermatozoa with a normal head size	Additional analysis proposal	Classification	ART possibility decision
Irregular head shape and multiple flagella	100 %	Not recommend	Screen for AURK mutations	Type I	Intracouple ART contraindicated
	<100 %	No euploid spermatozoa	Screen for AURK mutations	Type IIA	Intracouple ART contraindicated
		Presence of euploid spermatozoa	Screen for AURK mutations?	Type IIB	ICSI + PGD if available
Normal head shape and a single flagella	>10 %^a^	Normal level of aneuploid spermatozoa	Normal sperm chromatin condensation for spermatozoa with normal size head	Type IIIA	ICSI with spermatozoa with normal size head
		Normal level of aneuploid spermatozoa	Normal sperm chromatin condensation	Type IIIB	Intracouple ART
		High level of aneuploid spermatozoa	FISH	Type IIIC	ICSI + PGD if available

Classification of sperm with enlarged head according to light microscopy evaluation, percentage of spermatozoa with large heads, FISH studies, additional analysis performed, and ART (assisted reproductive technology). ^a^Guthauser et al., 2013 [26]

### Intracytoplasmic injection of morphologically selected spermatozoa (IMSI)

It has been suggested that IMSI can discriminate between normally shaped and abnormally shaped spermatozoa prior to microinjection. Our group showed that IMSI enabled the selection of haploid spermatozoa in semen sample from patients with MSHS and homozygous AURKC mutation (c.144delC). Unfortunately, a FISH analysis showed that none of the six selected spermatozoa was euploid [30]. Although motile sperm organelle morphology examinations (such as the IMSI-strict procedure) have not yet been tested in men with large-headed spermatozoa, we suggest that it might be able to exclude abnormal sperm in samples of semen with a low-to-moderate proportion of large-headed spermatozoa (Table 3).

### Preimplantation genetic diagnosis (PGD)

Kahraman et al. reported pregnancy rates of 33.3 % after 23 PGD cycles and 27.8 % after 60 non-PGD cycles. The implantation rate was higher in the PGD group (25.0 %) than in the non-PGD group (12.3 %). Forty six percent of the biopsied embryos were abnormal. One spontaneous abortion occurred in the PGD group (14.3 %), whereas seven of the 15 pregnancies obtained in the non-PGD group resulted in spontaneous abortion (46.7 %) [31].

### Flow cytometry

Flow cytometry is able to discriminate between spermatozoa that differ in size and granularity. Volume-based sorting was initially used to separate X- and Y-bearing bull spermatozoa, since slight differences in sperm DNA content are reflected in the sperm head volume [32]. Significant enrichment of Y-/X-sorted spermatozoa was achieved by coupling interferometry with flow cytometry in the absence of fluorescent staining [33]. Although the enrichment was not great enough to obtain sex-preselected offspring with a high degree of confidence, the technique might possibly be effective in men with large-headed spermatozoa. Indeed, some researchers have reported that the presence of large-headed spermatozoa is associated with a 3-fold relative increase in nuclear volume [13], which might be more easily detected and gated out using conventional flow cytometry (with or without interferometry).

To the best of our knowledge, stain-free flow cytometry techniques have not been used to sort viable spermatozoa in cases of patients with large-headed spermatozoa. However, flow cytometry with fluorescent DNA staining has already been used to isolate specific bovine and human sperm populations prior to FISH analysis [14, 34]. The combination of flow cytometry sorting with FISH may (i) reveal the presence of haploid spermatozoa with a normal head size that can be used for ICSI and (ii) provide an accurate prognosis in cases in which the spermatozoon head size is not uniform.

### Fish analysis

FISH analysis of raw semen (usually with three chromosome probes; see the photo in Table 2) is highly recommended when seeking to assess the feasibility of intracouple ICSI. In 1996, Yurov et al. analysed the semen of an infertile man in whom 40 % of the spermatozoa had abnormally large heads. The majority of spermatozoa with normal-sized heads were haploid and free from chromosomal aneuploidies, whereas most spermatozoa with large heads were diploid [11]. However, no pregnancies resulted from five ICSI cycles. In’t Veld et al. reported on a patient in whom all the spermatozoa had large heads and abnormal chromosome content [8]. A previous ICSI cycle had yielded a low fertilization rate (4 oocytes out of 15), two transfers and no pregnancies. The researchers suggested that a FISH analysis should always be used to evaluate the genetic risks. FISH analyses can also be performed on spermatozoa that would be selected and used for ICSI (i.e. spermatozoa with a normal-sized head, when available; see the photo in Table 2) [4, 7, 26].

### AURKC sequence analysis

This genetic analysis should be applied to patients with MSHS, whose spermatozoa have large heads, irregular shapes, multiple flagella and an abnormal chromosome content. In 2007, Dieterich et al. discovered that a high proportion of men with MSHS carried a homozygous mutation (c.144delC) in the Aurora kinase C (*AURKC*) gene [35]. In 2009, Dietrich et al. demonstrated that large-headed spermatozoa from *AURKC*-deficient patients were tetraploid - indicating that meiosis cannot be completed in the absence of functional AURKC [36]. These findings suggest that all spermatozoa from patients bearing AURKC mutations will have chromosome abnormalities and that ICSI should not be attempted. A comprehensive review of AURKC mutations and sperm DNA content in humans was published recently [37]. Lastly, AURKC sequence analysis should be performed in some ethnic groups; it has been demonstrated that the c.144delC homozygous AURKC mutation is the leading genetic cause of infertility in North African men [38], although the prevalence of heterozygosity seems to be five times lower in Tunisian males (0.4 %) than in men from North Africa as a whole (2 %) or in men from Morocco (1.7 %) [39].

### Karyotype

No specific abnormalities have been found in published cases of MSHS or large-headed spermatozoa. Although karyotyping and molecular testing for AZF microdeletions should be used for patients presenting with severe non-obstructive oligozoospermia, we consider that these analyses are not of value in this particular situation.

### Our classification proposition of enlarged sperm (Table 3)

We propose here a classification for semen containing large-headed spermatozoa: types I, IIA, IIB, IIIA, IIIB, IIIC and ART possibilities are summarized in the last column according to data of literature review developed in this article.

## Conclusion

Since the first case report of MSHS by Nistal et al. in 1977 [12], only 5 babies have been born to patients with large-headed spermatozoa enrolled in ART programmes. After additional analyses have been performed (as suggested in Table 2), genetic counselling may establish the likelihood of pregnancy with the patient’s spermatozoa (with or without PGD). The objectives of counselling are to (i) avoid unnecessary ICSI cycles when intracouple ART is contraindicated and (ii) estimate the usefulness of PGD/FISH when ICSI is possible. At last we propose a 6 types classification (I, IIA, IIB, IIIA, IIB, IIIC) based on sperm defects, associated with a ART possibility decision.

## Abbreviations

ART, assisted reproductive technology; AURKC, aurora kinase C; CASMA, computer-assisted sperm morphometry analysis; DNA, desoxyribonucleic acid; FISH, flourescence *in situ* hybrizydation; ICSI, intracytoplasmic sperm injection; IMSI, intracytoplasmic morphologically selected sperm injection; MSHS, macrocephalic sperm head syndrome; PGD, preimplanttion genetic diagnosis
